# Isolation of Smenopyrone, a Bis-γ-Pyrone Polypropionate from the Caribbean Sponge *Smenospongia aurea*

**DOI:** 10.3390/md16080285

**Published:** 2018-08-17

**Authors:** Germana Esposito, Roberta Teta, Gerardo Della Sala, Joseph R. Pawlik, Alfonso Mangoni, Valeria Costantino

**Affiliations:** 1 The NeaNat Group, Dipartimento di Farmacia, Università degli Studi di Napoli Federico II, via D. Montesano 49, 80131 Napoli, Italy; germana.esposito@unina.it (G.E.); roberta.teta@unina.it (R.T.); alfonso.mangoni@unina.it (A.M.); 2 Laboratory of Pre-clinical and Translational Research, IRCCS-CROB, Referral Cancer Center of Basilicata, 85028 Rionero in Vulture, Italy; gerardo.dellasala@crob.it; 3 Department of Biology and Marine Biology, University of North Carolina Wilmington, Center for Marine Science, 5600 Marvin K Moss Lane, Wilmington, NC 28409, USA; pawlikj@uncw.edu

**Keywords:** *Smenospongia aurea*, γ-pyrone, polypropionate, marine sponges, secondary metabolites, signaling molecules

## Abstract

The organic extract of the Caribbean sponge *Smenospongia aurea* has been shown to contain an array of novel chlorinated secondary metabolites derived from a mixed PKS-NRPS biogenetic route such as the smenamides. In this paper, we report the presence of a biogenetically different compound known as smenopyrone, which is a polypropionate containing two γ-pyrone rings. The structure of smenopyrone including its relative and absolute stereochemistry was determined by spectroscopic analysis (NMR, MS, ECD) and supported by a comparison with model compounds from research studies. Pyrone polypropionates are unprecedented in marine sponges but are commonly found in marine mollusks where their biosynthesis by symbiotic bacteria has been hypothesized and at least in one case demonstrated. Since pyrones have recently been recognized as bacterial signaling molecules, we speculate that smenopyrone could mediate inter-kingdom chemical communication between *S. aurea* and its symbiotic bacteria.

## 1. Introduction

**Figure 1:**
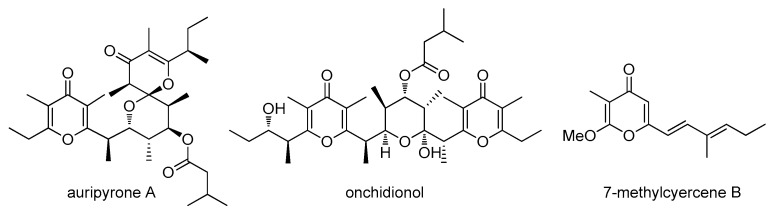
Some γ-pyrones of marine origin.

γ-Pyrones are a large class of biologically active compounds biosynthesized by polyketide synthases, multi-enzyme systems responsible for polyketide assembly through condensation of acyl-CoA units. They are mainly found in marine organisms [1] where they are thought to play a role as allomones or to be biosynthesized for defense purposes [2]. An important class that often contains one or more γ-pyrone rings are polypropionates, which are polyketides that mostly comprise propionate rather than acetate building blocks. In the marine environment, they are typically found in mollusks [3]. In 2013, Gavagnin et al. [4] reported a comprehensive study of polypropionates (e.g., onchnidionol, Figure 1) from marine pulmonate mollusks belonging to the family of *Onchidiidae* [5]. This family is known to produce a large array of C_32_ bis-γ-pyrones with cytotoxic properties at micromolar concentrations. Earlier in 1996, auripyrones A and B were isolated from the Japanese sea hare *Dolabella auricularia* [6], which is a species known for being the first source of dolastatin-10 [7]. A synthetic analogue of dolastatin-10 is one of the marketed drugs derived from a marine lead compound, which is the antibody drug-conjugate brentuximab vedotin commercialized under the name of Adcetris^®^.

**Figure 2:**
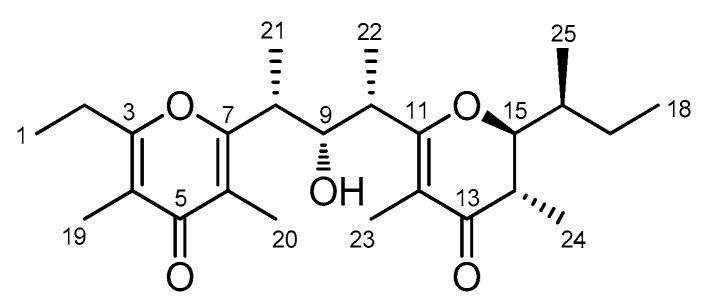
The structure of smenopyrone (**1**).

Among marine organisms, the sponges’ holobiome is one of the richest marine sources of secondary metabolites [8,9,10,11] and often provides skeletons with no counterpart in the terrestrial environment. However, no pyrone polypropionates have been isolated so far from a marine sponge. In this paper, we report on the isolation and structure elucidation of smenopyrone (**1**) (Figure 2), which is a bis-γ-pyrone compound, from a sample of *Smenospongia aurea*. The Caribbean sponges of the genus *Smenospongia* have been previously shown to contain a wide array of chlorinated metabolites deriving from the PKS-NRPS pathway such as smenamides, conulothiazoles, and smenothiazoles [12]. They have been used as guiding structures in our anti-cancer drug discovery programs [13,14].

## 2. Results

A sample of the sponge *Smenospongia aurea* was collected along the coast of Little Inagua (Bahamas Islands) in June 2013 at −15 m by experts of our group [15]. It was identified onboard of the vessel after the collection and it was immediately frozen and kept frozen until extraction. The sample (712 g wet weight) was extracted using our standard procedure involving extraction with MeOH/CHCl_3_ mixtures and partitioning between H_2_O and BuOH [16]. The total organic extract was chromatographed on a column packed with RP-18 silica gel. The fraction eluted with MeOH/H_2_O (9:1) was partitioned in a two-phase system composed of H_2_O (160 mL), MeOH (260 mL), CHCl_3_ (140 mL), and AcOH (5 mL). The organic layer was shown to contain known smenothiazoles and smenamides and one unknown compound. This fraction was subjected to repeated reversed-phase HPLC separations, which afford a fraction containing pure smenopyrone (**1**).

The [M + H]^+^ ion peak at *m*/*z* 419.2780 in the high-resolution ESI MS spectrum of **1** was indicative of the molecular formula C_25_H_38_O_5_, which corresponds to seven un-saturations. No olefinic proton was present in the in the ^1^H NMR spectrum while the ^13^C NMR spectrum showed the presence of eight non-protonated *sp*^2^ carbon atoms ranging from δ 109.2 to 197.9 (Table 1). Combined information from the DEPT spectrum and the molecular formula showed only one OH group to be present in the molecule. The remaining protons were linked to carbon atoms.

**Figure 3:**
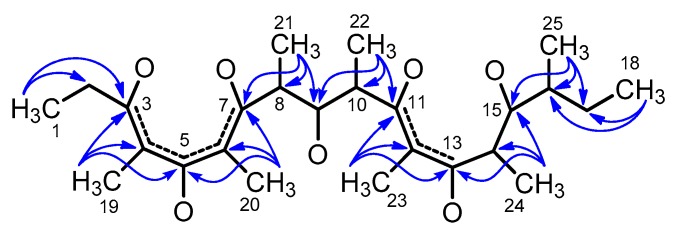
HMBC correlations (blue arrows) of methyl protons unequivocally defined the whole carbon skeleton of **1**. The π systems are depicted as dashed lines.

The proton NMR spectrum revealed the presence of nine methyl groups (two triplets, four doublets, and three singlets) out of 25 carbon atoms, which suggests a polypropionate structure. It has been shown that, for methyl rich compounds like **1**, the analysis of 2D NMR spectra can conveniently start from the HMBC spectrum [17]. As a result of the presence in **1** of nine methyl groups on alternated carbon atoms, the HMBC correlations of methyl protons provided enough information to build the whole carbon skeleton of **1** (Figure 3).

**Figure 4:**
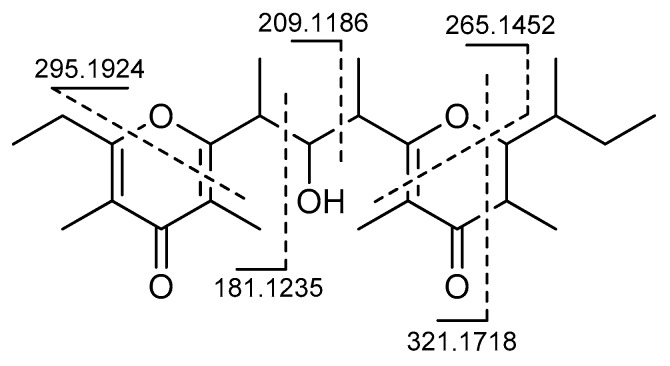
The main fragment peaks detected in the ESI tandem mass spectrum of smenopyrone (**1**).

The chemical shifts of the carbon atoms allowed the identification of two groups of contiguous *sp*^2^ carbon atoms, which are composed of five (C-3–C-7) and three (C-11–C-13) carbon atoms respectively. Among them, C-3, C-5, C-7, C-11, and C-13 were bonded to an oxygen atom, which is shown by their respective ^13^C chemical shifts. Two oxygen-bound *sp*^3^ carbon atoms (C-9 and C-15) were also present. The ^13^C chemical shift also suggested that the C-3–C-7 system is part of a pyrone because they fit well with the values reported for the similar tetrasubstituted pyrone system present in auripyrones A and B [6]. Likewise, the C-11–C-13 system (together with C-14 and C-15) was part of a tetrasubstituted dihydropyrone, which was suggested by a comparison of the chemical shift of **1** with those of the corresponding atoms of maurenone [18,19]. Therefore, the sole hydroxyl group in the molecule was linked to C-9. This completed the planar structure of **1**, which was further confirmed by the analysis of the fragment ions present in the tandem mass spectrum (Figure 4).

**Figure 5:**
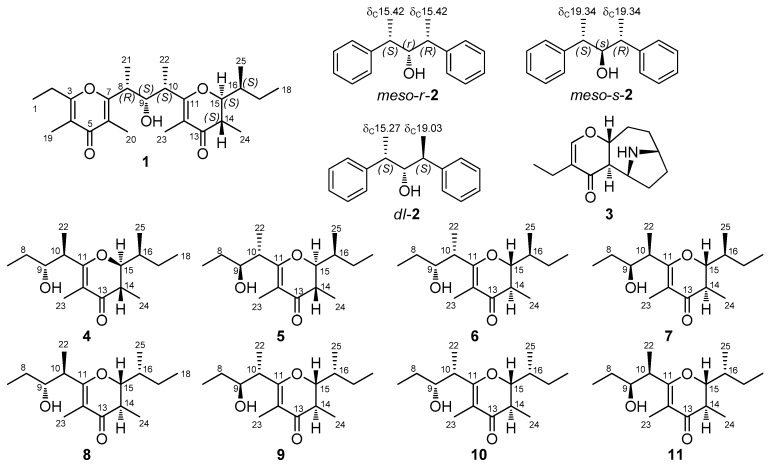
The three possible diastereomers of 2,4-diphenyl-3-pentanol (**2**), pinnamine (**3**), and the eight diastereomers of maurenone (**4**–**11**, the relative configuration of natural maurenone is as in **6**). These compounds were used as model compounds to elucidate the stereochemistry of smenopyrone (**1**). ^13^C NMR chemical shifts shown for **2** are adapted from Reference 20.

**Figure 6:**
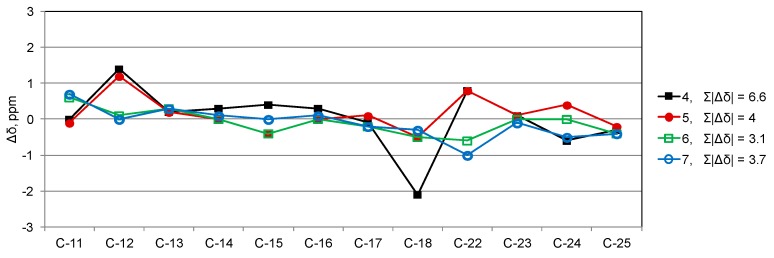
Difference in ^13^C NMR chemical shift (∆δ) between corresponding atoms of smenopyrone (**1**) and the four stereoisomers of the model compound maurenone (**4**–**7**). The sum of absolute values of ∆δ (Σ|Δδ|) was used to evaluate the overall fit between smenopyrone and **4**–**7**. Complete data can be found in Table S1 and Figure S1.

Determination of the stereochemistry of smenopyrone (**1**) was based on the following evidence. The large coupling constant between H-14 and H-15 showed them to be *trans*-axial and demonstrated the *trans* orientation of the groups at C-14 and C-15 (in the following discussion, we will assume the 14*S*,15*S* absolute configuration for these carbon atoms, which will eventually be proven correct). Configuration at C-9, C-10, and C-16 was based on a comparison of ^13^C NMR chemical shifts of smenopyrone (**1**) with those of the corresponding carbons of synthetic models, i.e., eight diastereomers of maurenone [19] (**4**–**11**, Figure 5). The chemical shift of C-25, which is very similar to that of compounds **4**–**7** but very far from that of compounds **8**–**11**, clearly demonstrated the *S* configuration at C-16. Configurations at C-9 and C-10 were based on an overall comparison with ^13^C NMR chemical shifts of model compounds, which were expressed as the sum of the absolute values of chemical shift differences, Σ|Δδ| (Table S1 and Figure 6 and Figure S1). The best fit (Σ|Δδ| = 3.1) was shown by model compound **6** and, therefore, the configuration was assigned as 9*S*,10*S*. The configuration at C-8 was assigned based on the observation that, in the isomers of the model compound 2,4-diphenyl-3-pentanol (**2**) (Figure 5), the ^13^C NMR chemical shifts of the methyl groups are remarkably different whether they are syn (δ_C_ ≈ 15) or anti (δ_C_ ≈ 19) to the OH group (Figure 5) [20]. This implied that C-21 and C-22 (both resonating at about δ 15) are both *syn* to the OH group at C-9 and, therefore, confirmed the relative configuration between C-9 and C-10 and determined the *R* configuration at C-8. 

The absolute configuration of smenopyrone was based on its ECD spectrum. Smenopyrone (**1**) contains two chromophores including a γ-dihydropyrone and a γ-pyrone. A *trans*-disubstituted γ-dihydropyrone as in **1** exists predominantly in the half-chair conformation with trans-diequatorial substituents (this is supported by the 12.8 Hz coupling constant between H-14 and H-15). In a half-chair, the enone system is slightly skewed and becomes an inherently chiral chromophore, which is expected to dominate the ECD spectrum. In contrast, a γ-pyrone is a planar, non-chiral chromophore and is expected to give a minor contribution to the overall ECD spectrum. The ECD curve recorded for smenopyrone (**1**) (Figure S2) showed a profile very similar to the ECD spectrum of the γ-dihydropyrone alkaloid pinnamine (**3**) [21], but also showed an opposite sign. This suggests that the γ-dihydropyrone moiety of **1** has the opposite configuration compared to pinnamine (**3**), which, therefore, defines the (8*R*,9*S*,10*S*,13*S*,14*S*,15*S*) absolute configuration for smenopyrone (**1**).

## 3. Discussion

Smenopyrone (**1**) is the first pyrone polypropionate isolated from a marine sponge. It contains a bis-γ-pyrone structure similar to that of auripyrones A and B, which were previously isolated from the Japanese sea hare *Dolabella auricularia*. These compounds are typical of some classes of marine mollusks but are also commonly found in terrestrial and marine fungi. It has been shown that polypropionates are biosynthesized through two entirely different pathways in mollusks and fungi even when the chemical structure of the synthesized compound is the same [22]. In mollusks, intact propionate units are introduced in the growing polyketide chain by incorporating methylmalonyl-CoA. In fungi, methyl branches are derived from methylation by *S*-adenosyl methionine (SAM). 

Recently, a series of closely related γ-pyrone polypropionates known as nocapyrones A-C and H-Q was shown to be produced by the bacterial symbiont *Nocardiopsis alba* CR167 isolated from the mollusk *Conus rolani* [23]. An iterative type-I polyketide synthase gene cluster has been identified as responsible for the biosynthesis of these compounds whose dipropionate backbone derives from condensation of two methylmalonyl CoA units. Additionally, it can be predicted that smenopyrone is assembled by an iterative type-I polyketide synthase through the condensation of one acetate unit and eight methylmalonyl-CoA units. The definite bacterial origin of nocapyrones suggests that other polypropionates from mollusks can be produced by bacteria and the same may hold true for sponge-derived smenopyrone. However, if nocapyrones are only occasionally found in *C. rolani* because *N. alba* is a casual symbiont of this mollusk, it is important to mention that the presence of smenopyrone (**1**) is by no means occasional in *S. aurea* because the compound has been consistently found in all specimens of Caribbean *S. aurea* we studied and is also present in the closely related species *S. conulosa*. Therefore, smenopyrone (**1**) is likely to be produced by obligate rather than casual sponge-symbionts.

These ideas also suggest an intriguing speculation about the role of smenopyrone in sponges. Sponges are sessile filter animals that harbor communities of microorganisms, which are either digested as a nutrient source or take part in a complex symbiotic relationship with the sponge. These host-associated bacteria make up 30% to 40% of sponge mass [24,25] and are often the real producer of the secondary metabolites isolated from the sponge extract. For example, in the case of the group of manzamine alkaloids, which is originally isolated from the sponge *Acanthostrongylophora ingens*, is subsequently shown to be produced by the associated actinomycete *Micromonspora* [26,27]. The idea of a possible inter-kingdom communication [28] between bacteria and sponges mediated by small molecules (e.g., lactones, which are regularly produced by many bacteria) is intriguing. It has been recently explored and demonstrated that, in the sponge *Suberites domuncula* [29,30], the *N*-3-oxodecanoyl-l-homoserine lactone can affect the expression of immune and apoptotic genes of the host and possibly enable the sponge to monitor bacterial community. Moreover, molecules affecting the quorum-sensing system (the system of bacteria use to communicate) were found in sponge extracts [31], which suggests the possibility of complex communications and interactions between the two kingdoms.

Endogenous pyrone-containing polyketides have been recently identified as the signaling molecules of a previously orphan signal system of proteobacteria [32]. It is, therefore, exceedingly easy to speculate that smenopyrone may similarly function as a signaling molecule and mediate the communication between obligate symbiotic bacteria of *S. aurea* and the host sponge. While this is merely a fascinating speculation at present, it is worth pursuing this hypothesis in order to determine if, in fact, smenopyrone or related pyrone polypropionates act as inter-kingdom signaling molecules for bacterial symbionts of sponges or mollusks.

## 4. Materials and Methods

### 4.1. General Experimental Procedure

ECD spectra were recorded using a Jasco-715 spectropolarimeter (Easton, MD, USA). NMR spectra were determined on Varian Unity Inova spectrometers at 700 MHz and 500 MHz. Chemical shifts were referenced to the residual solvent signal (CD_3_OD: δ_H_ 3.31, δ_C_ 49.0; CDCl_3_: δ_H_ 7.26, δ_C_ 77.0). For an accurate measurement of the coupling constants, the one-dimensional ^1^H NMR spectra were transformed at 128 K points (digital resolution < 0.1 Hz). The HSQC spectra were optimized for ^1^*J*_CH_ = 142 Hz and the ^13^C HMBC experiments for ^2,3^*J*_CH_ = 8.3 Hz. High-resolution ESI-MS and MS/MS experiments were performed on a Thermo LTQ Orbitrap XL mass spectrometer (Thermo Fisher Scientific Spa, Rodano, Italy) coupled to a Thermo U3000 HPLC system (Agilent Technology, Cernusco sul Naviglio, Italy). High performance liquid chromatography (HPLC) separations were achieved on an Agilent 1260 Infinity Quaternary LC apparatus (Agilent Technology, Cernusco sul Naviglio, Italy) equipped with a Diode-Array Detector (DAD).

### 4.2. Collections, Extraction, and Isolation

A specimen of *Smenospongia aurea* (712 g wet weight) was collected on the 9 July 2013 at depths of 15 m by scuba diving along the coast of Great Inagua (Bahamas Islands, 21°03′24.55′′ N–73°25′27.76′′ W). The collected sample was a relatively small portion of a much larger sponge and was excised with a sharp scalpel to minimally affect the remaining sponge tissue and allow recovery and regrowth. After collection, the sample was unambiguously identified onboard using a web-based photographic and taxonomic key, known as The Sponge Guide (www.spongeguide.org), with subsequent confirmation by sponge taxonomist Sven Zea. The sample was frozen immediately after collection and stored at −20 °C until extraction. A voucher specimen of the organism is stored at Dipartimento di Farmacia, “TheBlueChemistryLab” laboratory, Università degli Studi di Napoli “Federico II” with the reference number 06/07/13.

To perform the present study, the sample was allowed to reach room temperature, then cut in small pieces, homogenized, and extracted following our standard procedure [33]. The total organic extract (16.31 g) was chromatographed on a column packed with RP-18 silica gel. The fraction was eluted with MeOH/H_2_O (9:1, 363.7 mg) and was partitioned in a two-phase system composed of H_2_O (160 mL), MeOH (260 mL), CHCl_3_ (140 mL), and AcOH (5 mL). The organic layer, which contained smenothiazoles [34], smenamides [35], and smenopyrone (**1**), was subjected to reversed-phase HPLC separation (column 250 × 10 mm, 10 μm, Luna (Phenomenex) C18, eluent A: H_2_O, eluent B: MeOH, gradient: 55→100% B, over 60 min, flow rate 5 mL min^−1^), which affords a fraction (t_R_ = 29 min) containing compound **1**. The former fraction was subjected to new separations on reversed-phase HPLC (column 250 × 4.6 mm, 5 μm, Luna (Phenomenex) C18, eluent A: H_2_O, eluent B: ACN, gradient: 50→100% B, over 35 min, flow rate 1 mL min^−1^), which gave 12 µg of a pure compound **1** (t_R_ = 16 min).

Smenopyrone (**1**): Colorless glass, UV (MeOH): λ_max_ (ε) 260 (21,600), ECD (MeOH): λ_max_ (Δε) 306 (+1.2), 277 (−5.5), HR-ESI-MS (High Resolution-ElectroSpray Ionization-Mass Spectrometry) (positive ion mode, MeOH) *m*/*z* 419.2780, [M + H]^+^ (C_25_H_39_O_5_^+^ gives 419.2792, Δ − 2.8 ppm), ^1^H NMR (CDCl_3_): 3.99 (dd, *J* = 8.0 and 4.6 Hz, H-9), 3.84 (m, H-15), 3.07 (dq, *J* = 8,0 and 7.0 Hz, H-8), 2.92 (dq, *J* = 4.6 and 7.0 Hz, H-10), 2.71 (q, *J* = 7.6 Hz, H_2_-2), 2.51 (dq, *J* = 12.4 and 3.2 Hz, H-14), 1.94 (s, H_3_-20), 1.94 (s, H_3_-19), 1.76 (m, H-16), 1.67 (s, H_3_-23), 1.62 (m, H-17a), 1.26 (m, H-17b), 1.24 (d, *J* = 7.0 Hz, H_3_-22), 1.24 (d, *J* = 7.0 Hz, H_3_-21), 1.22 (t, *J* = 7.6 Hz, H_3_-1), 1.09 (d, *J* = 6.9 Hz, H_3_-24), 1.05 (d, *J* = 6.9 Hz, H_3_-25), 0.94 (t, *J* = 7.4, H_3_-18); ^13^C NMR (CDCl_3_): 195.4 (C, C-13), 179.5 (C, C-5), 172.4 (C, C-11), 164.0 (C, C-3), 164.7 (C, C-7), 118.2 (C, C-4), 119.4 (C, C-6), 108.5 (C, C-12), 87.4 (CH, C-15), 75.1 (CH, C-9), 40.6 (CH, C-14), 38.6 (CH, C-10), 38.3 (CH, C-8), 35.1 (CH, C-16), 26.2 (CH_2_, C-2), 22.1 (CH_2_, C-17), 16.5 (CH_3_, C-25), 15.3 (CH_3_, C-21), 13.9 (CH_3_, C-22), 12.2 (CH_3_, C-18), 11.6 (CH_3_, C-1), 10.6 (CH_3_, C-24), 9.8 (CH_3_, C-20), 9.4 (CH_3_, C-19), 9.3 (CH_3_, C-23), ^1^H, and ^13^C NMR (CD_3_OD): Table 1. 

## Figures and Tables

**Table 1:** NMR Data of smenopyrone (**1**) (^1^H 700 MHz, ^13^C 175 MHz, CD_3_OD).

Position	δ_C_, Type		δ_H_, Mult (*J* in Hz)	HMBC ^a^
1	11.7 (CH_3_)		1.25 (t, 7.5)	2, 3
2	25.7 (CH_2_)	a,b	2.71 (m)	1, 3
3	167.5 (C)		-	
4	119.0 (C)		-	
5	182.0 (C)		-	
6	120.3 (C)		-	
7	167.7 (C)		-	
8	41.7 (CH)		3.15 (quintet, 7.1)	9, 21
9	75.6 (CH)		4.02 (t, 7.1)	7, 8, 21, 22
10	41.5 (CH)		2.93 (quintet, 7.0)	9, 11, 22
11	175.6 (C)		-	
12	109.2 (C)		-	
13	197.9 (C)		-	
14	41.5 (CH)		2.53 (dq, 12.8, 6.9)	13, 15, 24
15	88.0 (CH)		3.84 (dd, 12.8, 3.0)	
16	36.6 (CH)		1.78 (m)	
17	23.0 (CH_2_)	a	1.64 (m)	
		b	1.29 (m)	
18	12.1 (CH_3_)		0.98 (t, 7.5)	16, 17
19	9.6 (CH_3_)		1.93 (s)	3, 4, 5
20	10.1 (CH_3_)		1.91 (s)	5, 6, 7
21	15.5 (CH_3_)		1.28 (d, 7.1)	7, 8, 9
22	14.1 (CH_3_)		1.26 (d, 6.9)	9, 10, 11
23	9.4 (CH_3_)		1.63 (s)	11, 12, 13
24	10.7 (CH_3_)		1.06 (d, 6.9)	13, 14, 15
25	16.6 (CH_3_)		1.11 (d, 6.9)	15, 16, 17

^a^ HMBC correlations from proton stated to the indicated carbon.

## Supplementary Materials

The following are available online at http://www.mdpi.com/1660-3397/16/8/285/s1, Table S1: ^13^C NMR chemical shift of smenopyrone **1** compared with those of the eight diastereomers of maurenone **4**–**11**, Figure S1: Difference in ^13^C NMR chemical shift between corresponding atoms of **1** and **4**–**11**, Figure S2: UV and ECD spectra of **1**, Figure S3: High-resolution ESI mass spectrum of **1**, Figure S4: High-resolution ESI MS/MS spectrum (parent ion at m/z 419.28) and fragmentation of **1**, Figures S5–S10: ^1^H-NMR, COSY, NOESY, HSQC, and HMBC spectra of **1** (700 MHz, CD_3_OD).
