# Supplementary material for: Isolation of Smenopyrone, a Bis-γ-Pyrone Polypropionate from the Caribbean Sponge Smenospongia aurea
Source: Mar Drugs. 2018 Aug 17;16(8):285. doi: 10.3390/md16080285 (PMC6117678; doi:10.3390/md16080285)
Supplement: Supplementary file 1 [file marinedrugs-16-00285-s001.zip › marinedrugs-342181-SI.pdf]

Supplementary Information

Table S1. ^{13}C NMR chemical shift of smenopyrone (**1**) in $CDCl_3$ compared with those of the eight diastereomers **4-11** of maurenone.
Figure S1. Difference in ^{13}C NMR chemical shift ($\Delta\delta$) between corresponding atoms of smenopyrone (**1**) and the eight stereoisomers **4-11** of the model compound maurenone.
Figure S2. UV and ECD spectra of smenopyrone (**1**).
Figure S3. Positive ion mode high-resolution ESI mass spectrum of smenopyrone (**1**).
Figure S4. Positive ion mode high-resolution ESI MS/MS spectrum (parent ion at *m/z* 419.28) and fragmentation of smenopyrone (**1**).
Figure S5. ^{1}H-NMR spectrum of smenopyrone (**1**) (700 MHz, CD_3OD).
Figure S6. COSY spectrum of smenopyrone (**1**) (700 MHz, CD_3OD).
Figure S7. NOESY spectrum of smenopyrone (**1**) (700 MHz, CD_3OD).
Figure S8. HSQC spectrum of smenopyrone (**1**) (700 MHz, CD_3OD).
Figure S9. HMBC spectrum of smenopyrone (**1**) (700 MHz, CD_3OD).
Figure S10. Expansion of methyl region of the HMBC spectrum of smenopyrone (**1**) (700 MHz, CD_3OD).

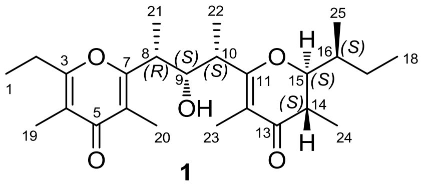

1

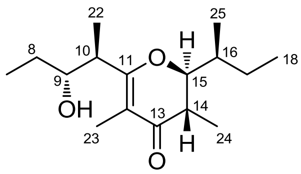

4

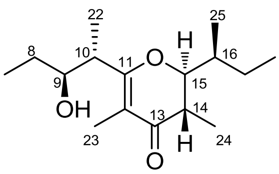

5

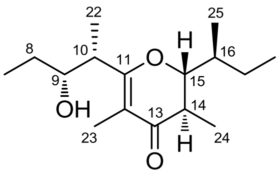

6

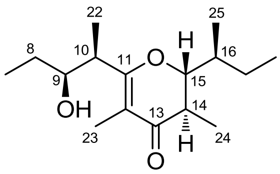

7

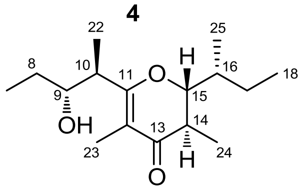

8

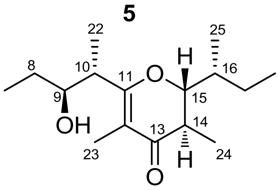

9

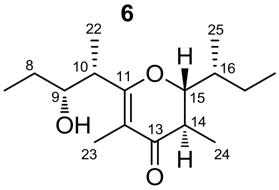

10

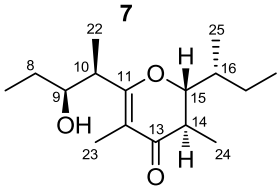

11

Table S1. ^{13}C NMR chemical shifts of smenopyrone (**1**) in $CDCl_3$ compared with those of the eight diastereomers of maurenone, **4**-**11**.

Pos.		δ_C ($CDCl_3$)								
		**1**	**4**	**5**	**6**	**7**	**8**	**9**	**10**	**11**
9	CH	75.1	75.5	75.4	75.1	74.8	75.3	75.3	74.8	75.2
10	CH	38.6	41.1	41.4	41.6	41.5	41.2	41.2	41.5	41.8
11	C	172.4	172.4	172.3	173.0	173.1	172.6	172.3	173.2	173.2
12	C	108.5	109.9	109.7	108.6	108.5	109.8	109.8	108.6	108.7
13	C	195.4	195.6	195.6	195.7	195.7	195.5	195.5	195.7	195.7
14	CH	40.6	40.9	40.6	40.6	40.7	40.5	40.6	40.7	40.5
15	CH	87.4	87.8	87.0	87.0	87.4	84.5	84.5	84.7	84.2
16	CH	35.1	35.4	35.1	35.1	35.2	35.6	35.6	35.5	35.6
17	CH_2	22.1	22.0	22.2	21.9	21.9	26.8	26.8	26.5	26.5
18	CH_3	12.2	10.1	11.7	11.7	11.9	9.9	11.9	10.2	11.8
22	CH_3	13.9	14.7	14.7	13.3	12.9	14.6	14.6	12.9	13.4
23	CH_3	9.3	9.4	9.4	9.3	9.2	9.4	9.4	9.2	9.3
24	CH_3	10.6	10.0	11.0	10.6	10.1	9.9	9.9	9.5	9.7
25	CH_3	16.5	16.2	16.3	16.1	16.1	12.3	12.3	12.4	12.3
$\Sigma\|\Delta\delta\|$			***6.6***	***4.0***	***3.1***	***3.7***	***17.8***	***15.6***	***17.1***	***15.5***

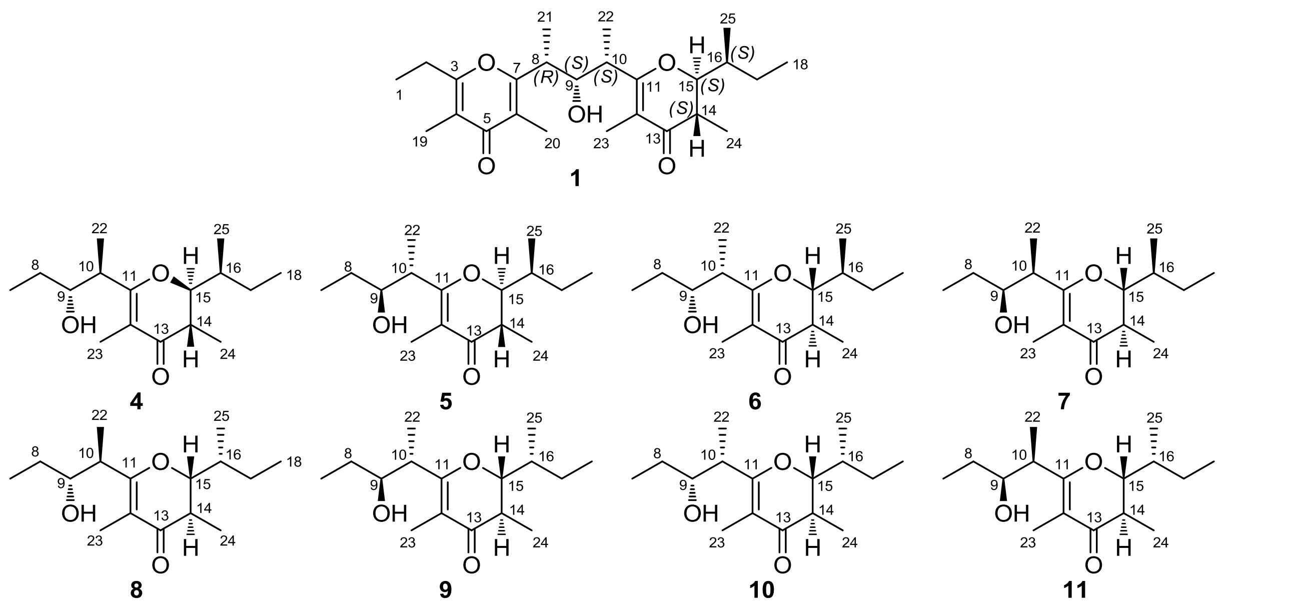

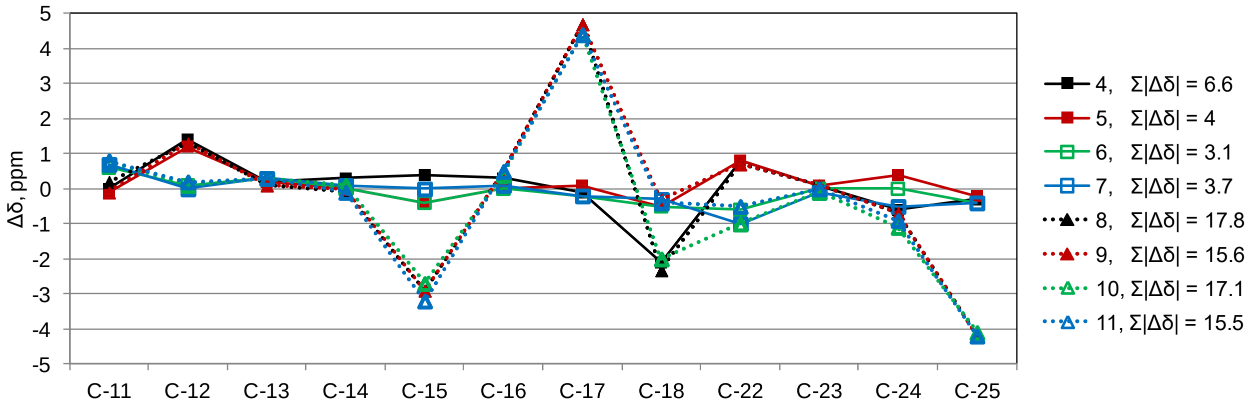

Figure S1. Difference in ^{13}C NMR chemical shift (Δδ) between corresponding atoms of smenopyrone (**1**) and the eight stereoisomers **4**-**11** of the model compound maurenone. The sum of absolute values of Δδ (Σ|Δδ|) was used to evaluate the overall fit between **1** and **4**-**11**.

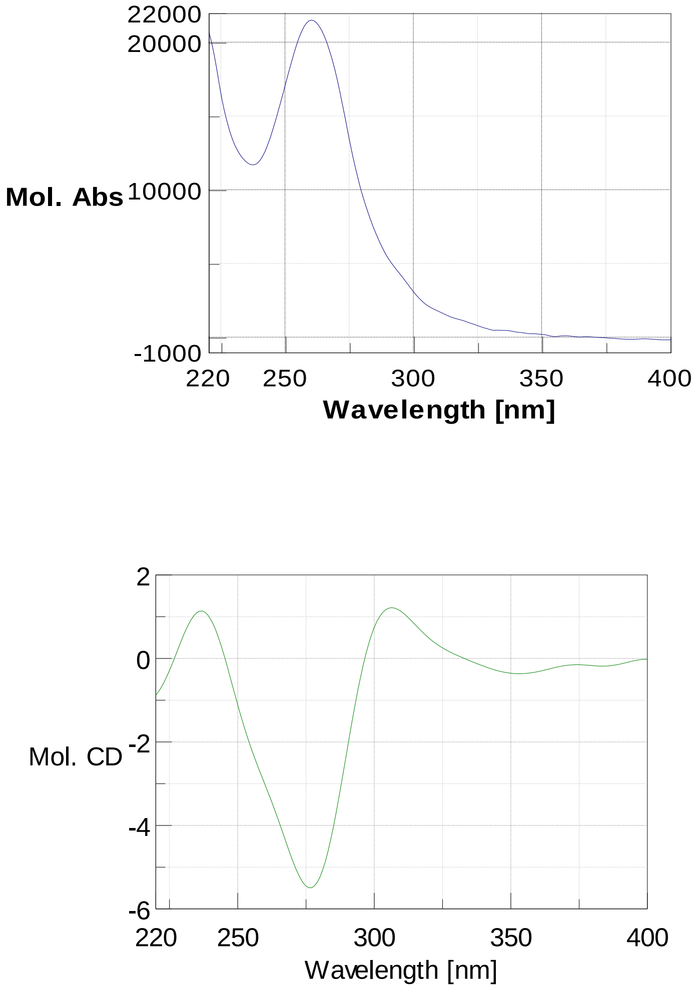

Figure S2. UV (top) and ECD (bottom) spectra (MeOH) of smenopyrone (**1**).

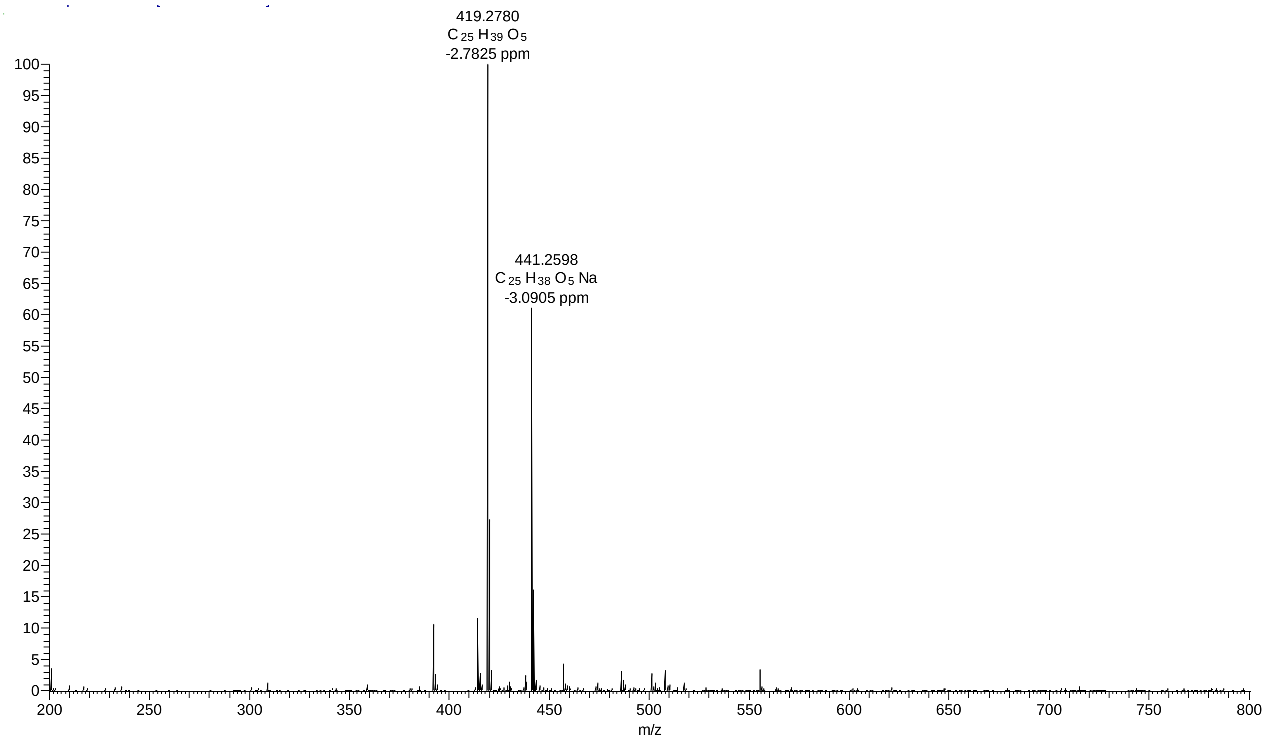

Figure S3. Positive ion mode high-resolution ESI mass spectrum of smenopyrone (**1**).

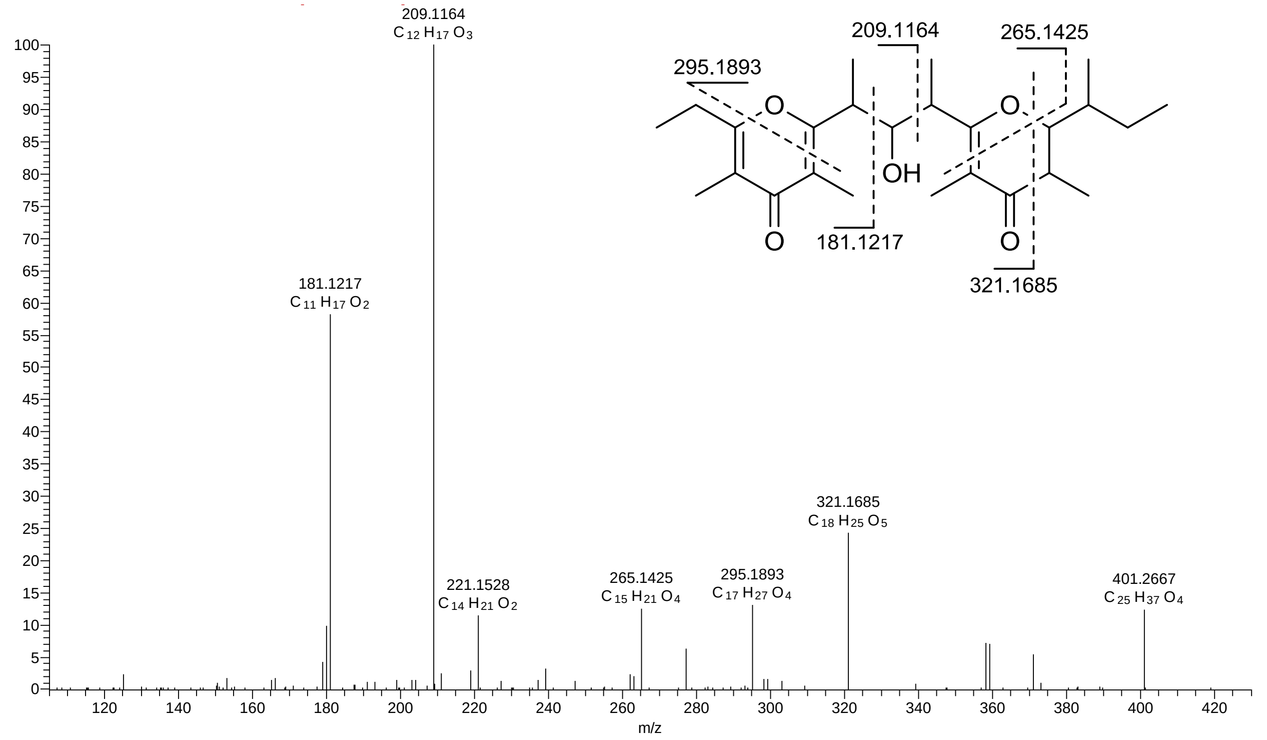

Figure S4. Positive ion mode high-resolution ESI MS/MS spectrum (parent ion at *m/z* 419.28) and fragmentation of smenopyrone (**1**).

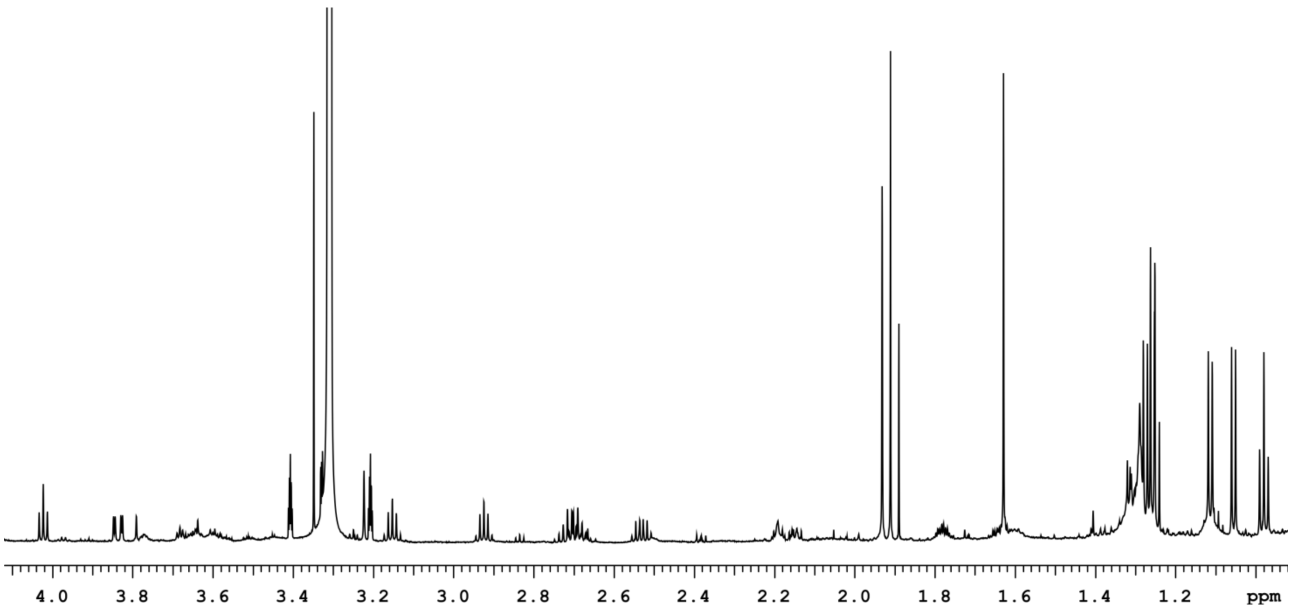

Figure S5. ^{1}H-NMR spectrum of smenopyrone (**1**) (700 MHz, CD_3OD)

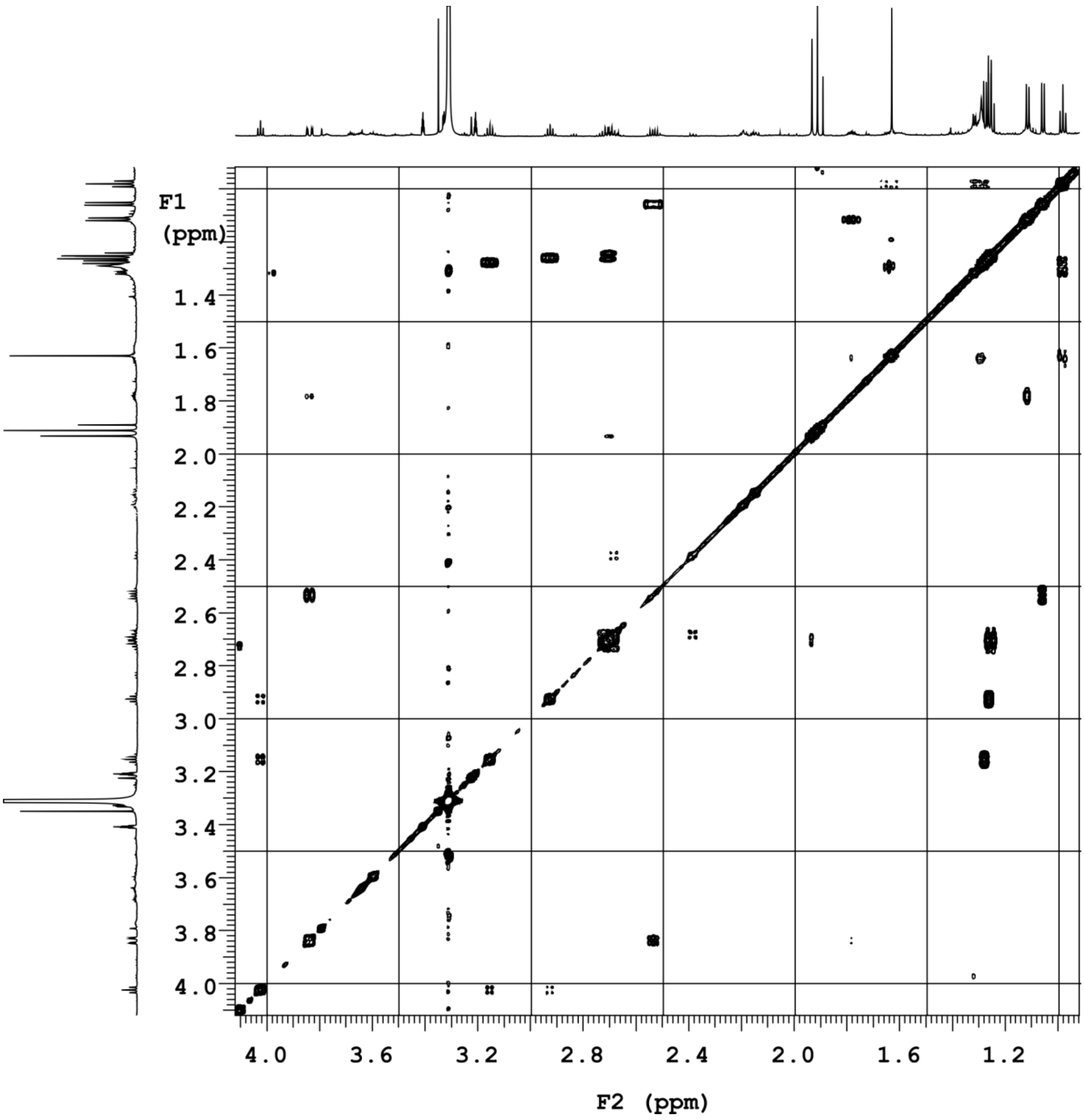

Figure S6. COSY spectrum of smenopyrone (**1**) (700 MHz, CD_3OD)

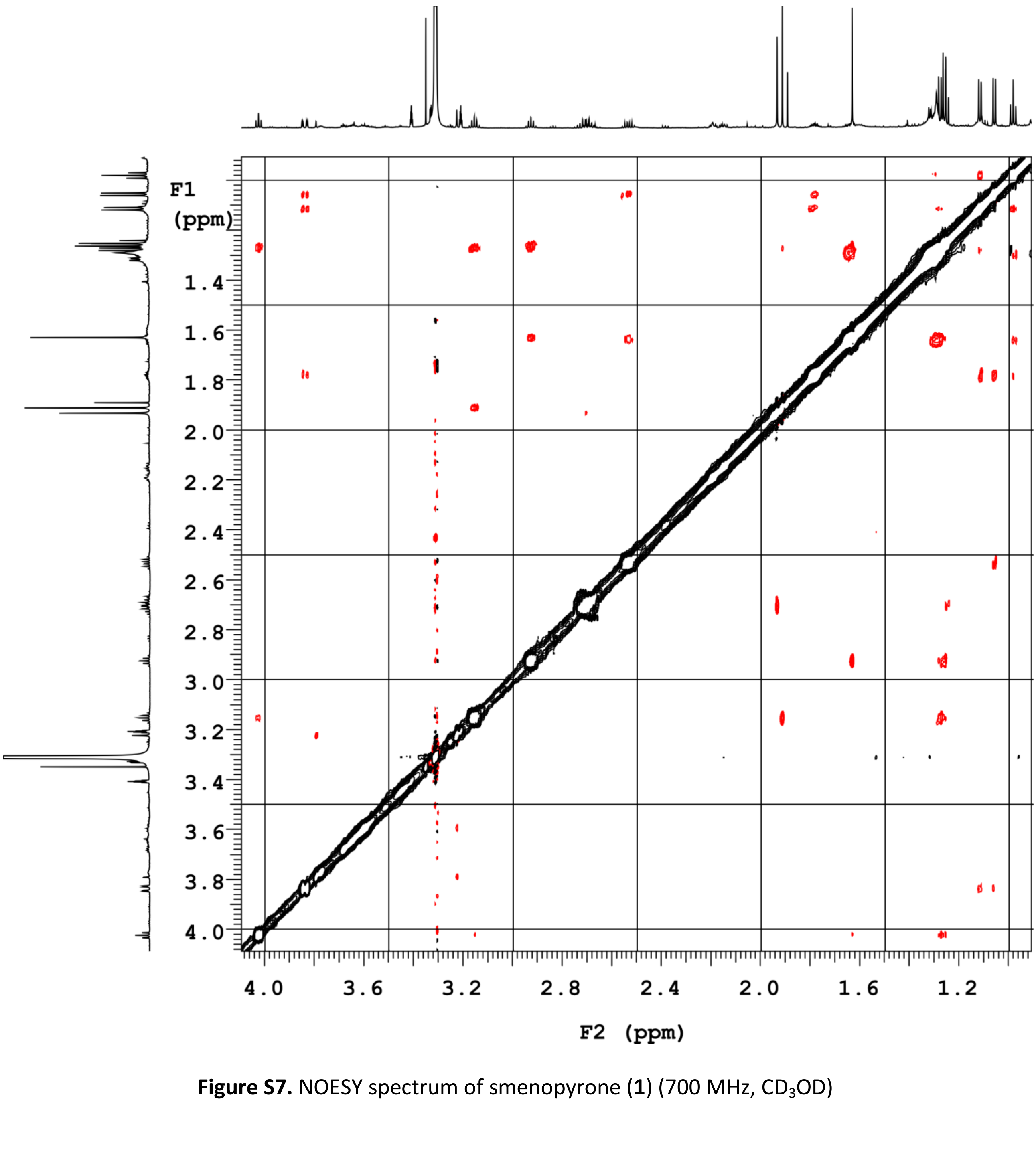

Figure S7. NOESY spectrum of smenopyrone (**1**) (700 MHz, CD_3OD)

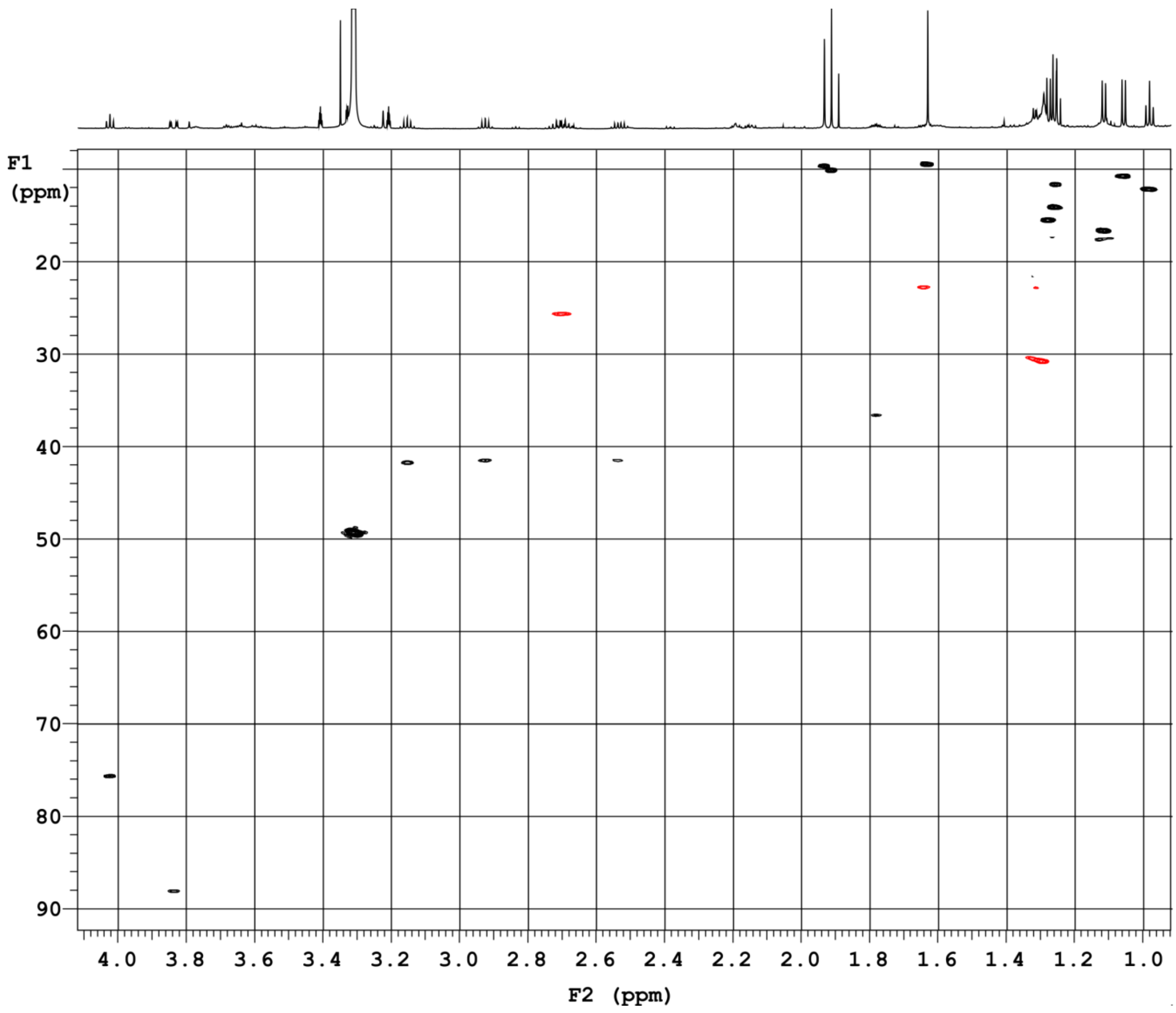

Figure S8. HSQC spectrum of smenopyrone (**1**) (700 MHz, CD_3OD)

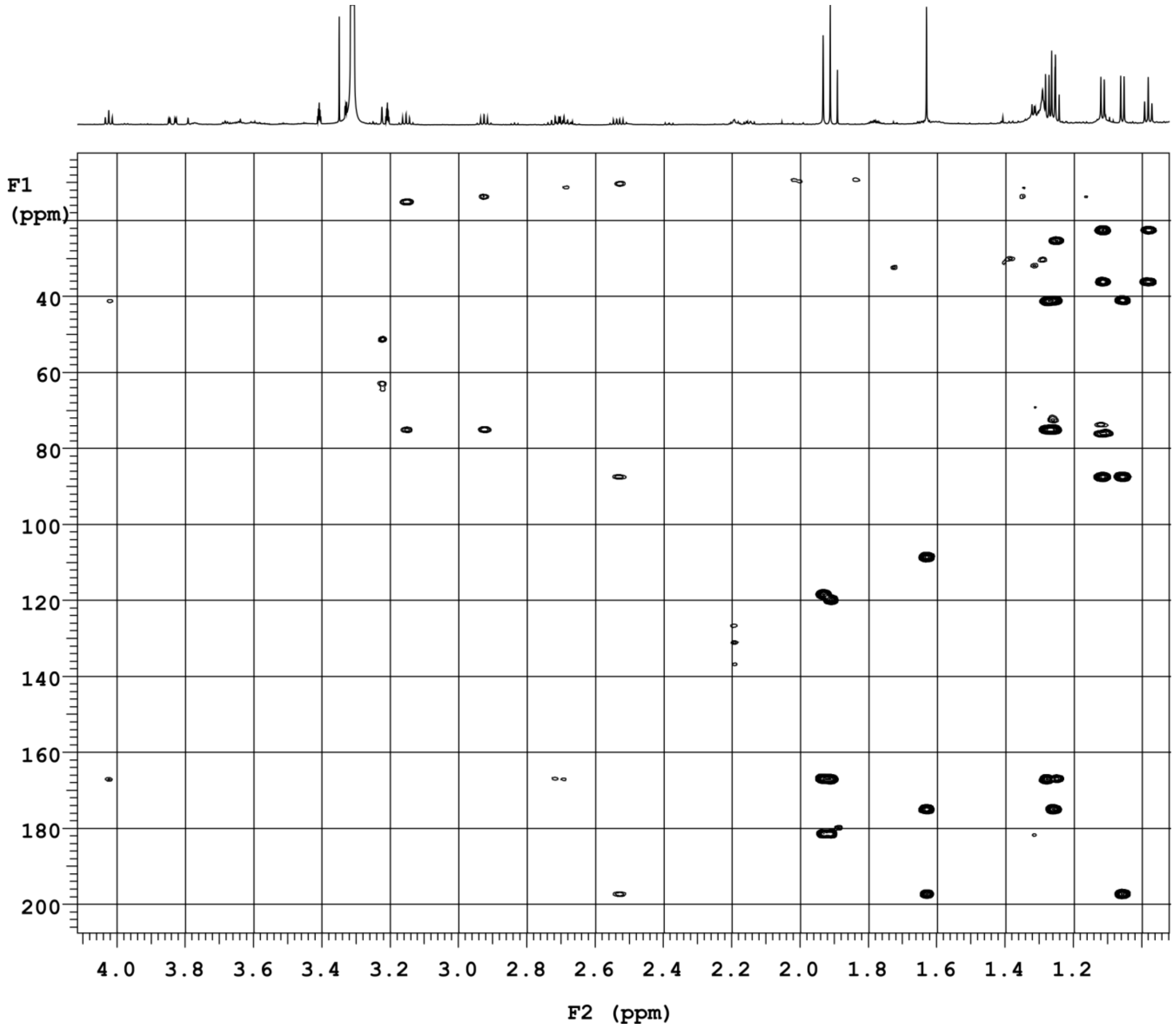

Figure S9. HMBC spectrum of smenopyrone (**1**) (700 MHz, CD_3OD)

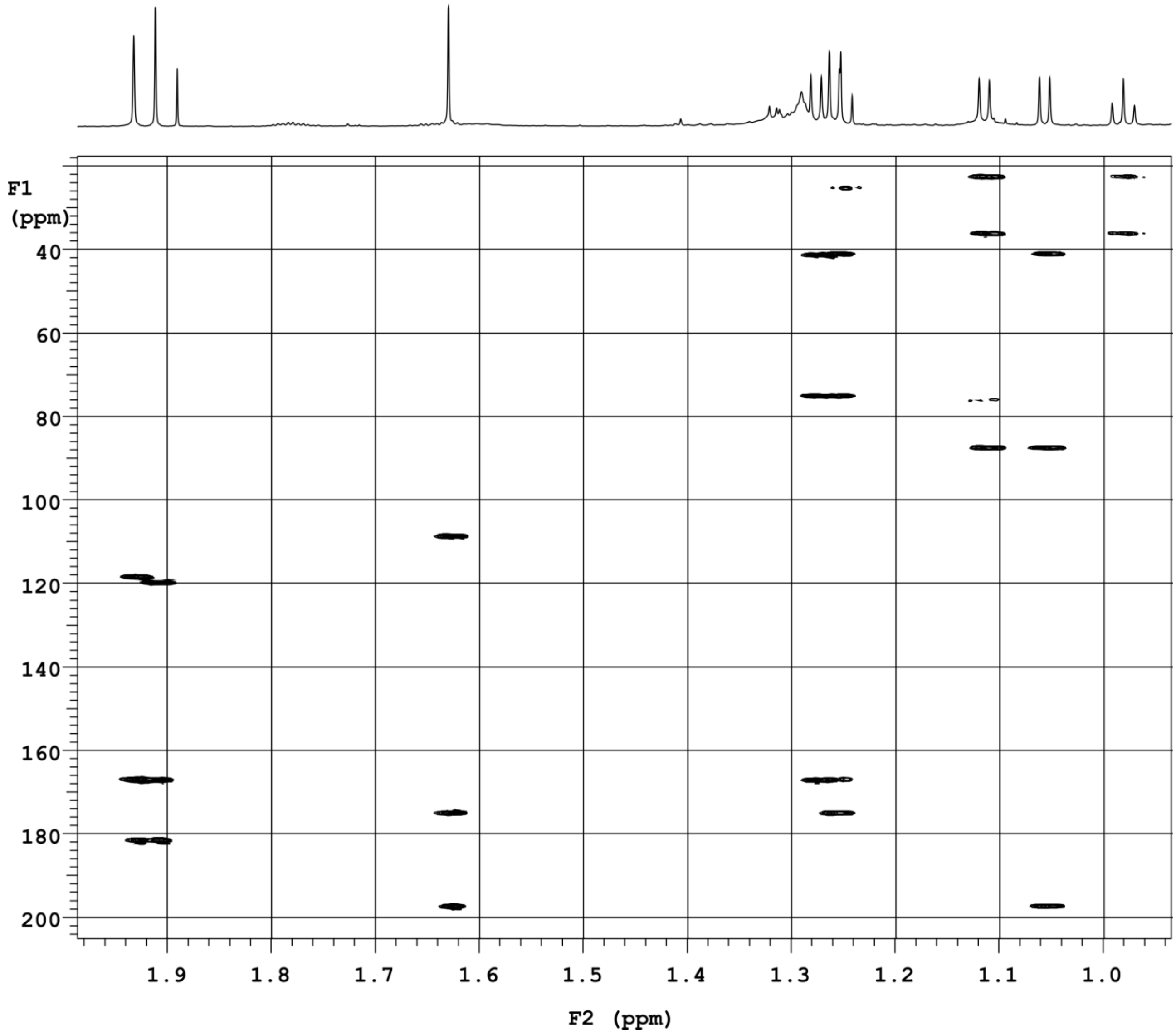

Figure S10. Expansion of methyl region of the HMBC spectrum of smenopyrone (**1**) (700 MHz, CD_3OD)
